# Supplementary material for: Laser-driven x-ray and proton micro-source and application to simultaneous single-shot bi-modal radiographic imaging
Source: Nat Commun. 2020 Dec 2;11:6174. doi: 10.1038/s41467-020-19838-y (PMC7710721; doi:10.1038/s41467-020-19838-y)
Supplement: Supplementary file 1 — Supplementary Information [file 41467_2020_19838_MOESM1_ESM.pdf]

SUPPLEMENTARY INFORMATION

Laser-driven x-ray and proton micro-source and application to simultaneous single-shot bi-modal radiographic imaging

T.M. Ostermayr, C. Kreuzer, F.S. Englbrecht, J. Gebhard, J. Hartmann, A. Huebl, D. Haffa, P. Hilz, K. Parodi, J. Wenz, M.E. Donovan, G. Dyer, E. Gaul, J. Gordon, M. Martinez, E. Mccary, M. Spinks, G. Tiwari, B.M. Hegelich, and J. Schreiber

Supplementary Note 1: Proton spectrum

This supplementary note shows proton spectra for several shots in supplementary figure 1 and a linear plot for one example spectrum in supplementary figure 2. This demonstrates the correlation of the spectra measured towards the side within single shots, the reproducibility of peaked spectra, and the narrow spectral bandwidth of the source.

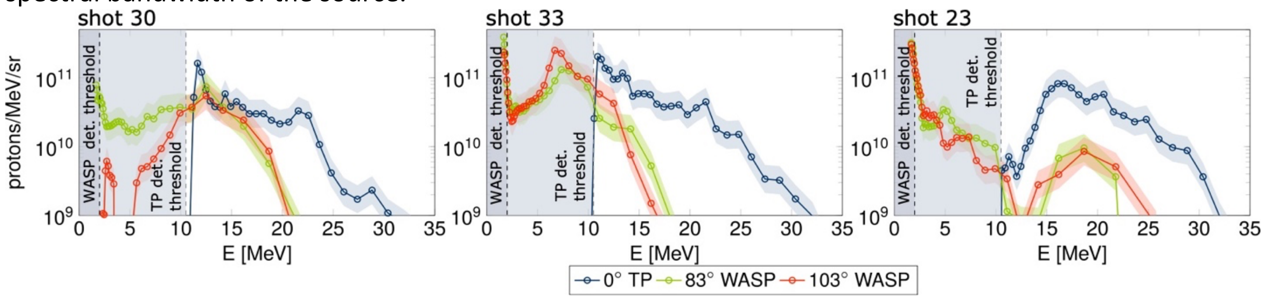

Supplementary Figure 1 | Proton spectrum in multiple shots. *Proton spectra measured in different angles along the horizontal (details see main text) for different shots on micro-needle targets. Shot-to-shot fluctuations are evident, but the spectral shape and correspondence between 83 degree and 103 degree emission are consistent. Error bands are an estimate of absolute accuracy including detector calibration.*

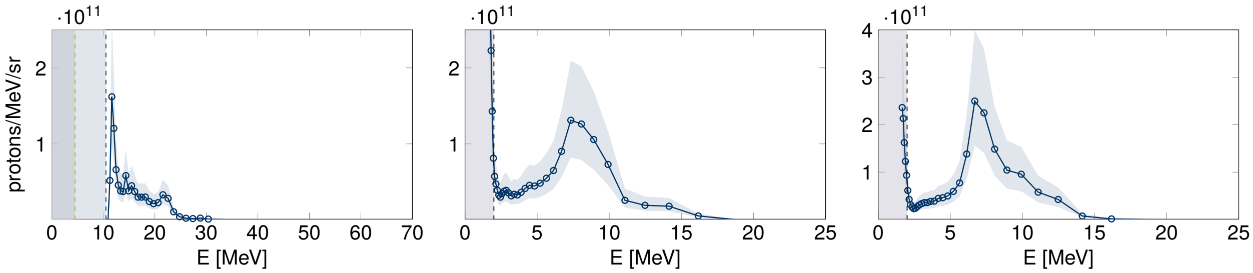

Supplementary Figure 2 | Linear proton spectrum plot. *Linear plots for the proton spectra in shot 33 as measured in the Thomson parabola (left), in the 83 degree wide angle spectrometer (center) and in the 103 degree wide angle spectrometer (right), showing strongly peaked spectra towards both sideward spectrometers. Error bands are an estimate of absolute accuracy including detector calibration.*

Supplementary Note 2: Effective source size

Setup and simulation: In experiments, the needle was first aligned with the silicon surface plane using a laser diode. It was then displaced by 50-100 um normal to the silicon-plane in order to ensure that the second (distal) edge of the silicon would create the shadow for the edge-spread function measurement. Thereby, this small apparent 'misalignment' allowed for the unambiguous definition of magnification (M=D/L) by exactly defining the edge position. A schematic of the setup is shown in supplementary figure 3a.

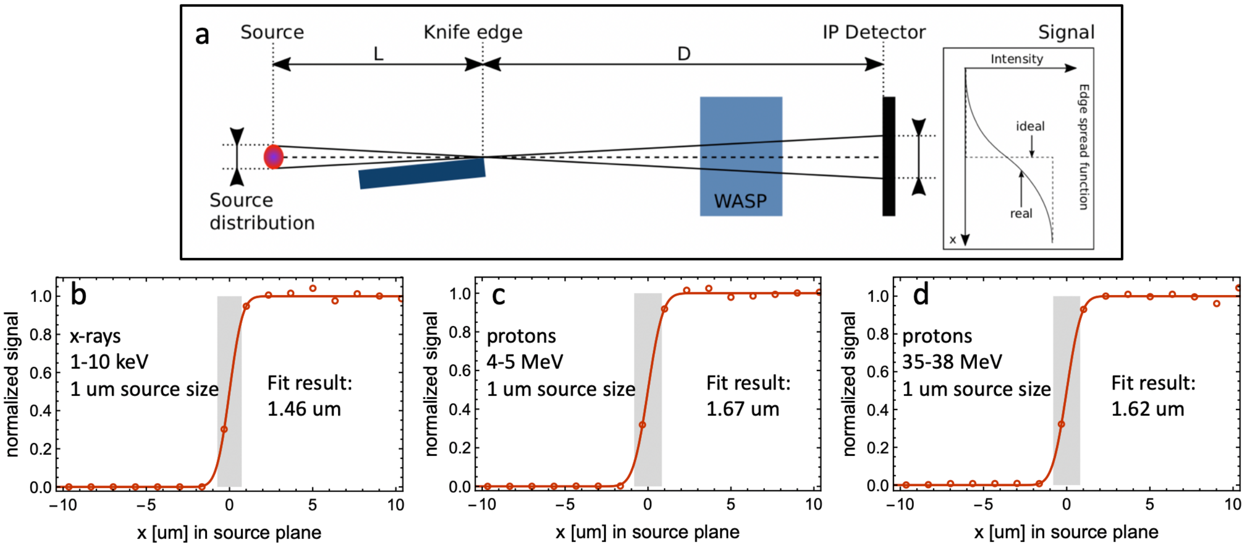

Supplementary Figure 3 | FLUKA simulations of source size measurement. *a: setup sketch and measurement idea via edge spread function. b: FLUKA simulation for 1-10 keV x-rays from a uniform 1 um source with 50 um offset between needle and silicon surface plane. The points are simulated data, red line is the erf-fit, gray area indicates the retrieved FWHM size. c: same setup with 4-5 MeV protons. d: same setup with 35-38 MeV protons. Figure a reproduced with permission from "Relativistically Intense Laser Microplasma Interactions" by Tobias Ostermayr, Springer Thesis, (2019).*

FLUKA simulations of this setup were performed for x-rays (1-10 keV range) and protons (4-5 MeV and 35-38 MeV ranges) for a lateral offset of 50 um between needle and silicon surface (supplementary figure 3b-d). The simulated source distribution had a 1 um diameter with uniform/isotropic emission. All distances were chosen comparable to our experiments and a 25 um detector resolution was used. Instead of simulating a WASP, we simulated two separate energy bands for protons.

Source-size analysis was done in analogy to experimental data, using an erf-fit. In all cases the original 1 um source is overestimated by the retrieved FWHM size, but comes out still well below 2 um. Parts of the overestimation can be attributed to the simulated uniform source distribution, while the erf-fit assumes an underlying Gaussian LSF/PSF. Another important factor is the limited (but realistic) detector resolution; with about 20-times magnification and 25 um detector pixel size, the effective detector resolution corresponds to only 1.25 um (source size)/px.

Meanwhile, the data presented in supplementary figure 3b-d do not show indications of a 'non-black' edge. Signal levels in the shadow are small for all cases, and the shadow does extend up to the signal rise that is due to the geometrical edge. In other words, in none of the cases significant signal was observed in the geometric shadow (which would complicate measurements).

E.g., traces for 4-5 and 35-38 MeV look comparable, which indicates that higher energy protons still cannot penetrate the edge in significant numbers.

Given these simulations, the method was expected to sufficiently resolve sources in the 1+ um range, and that smaller source-sizes were unexpected for this experiment.

Experiments: From lineouts (edge spread function) to effective source size. Supplementary figure 4 shows an example of raw data and lineouts taken for the ESF measurements. Supplementary figure 5 shows an example for the ESF measurement and fit for x-rays. Supplementary figure 6 shows one such measurement and compares with analytical calculations for different source sizes including diffraction on the knife edge. Finally, supplementary figure 7 shows the ESF measurement and fit for the protons. Full widths at half maximum values were extracted from the fits for each measurement and reported in figure 1d of the main text.

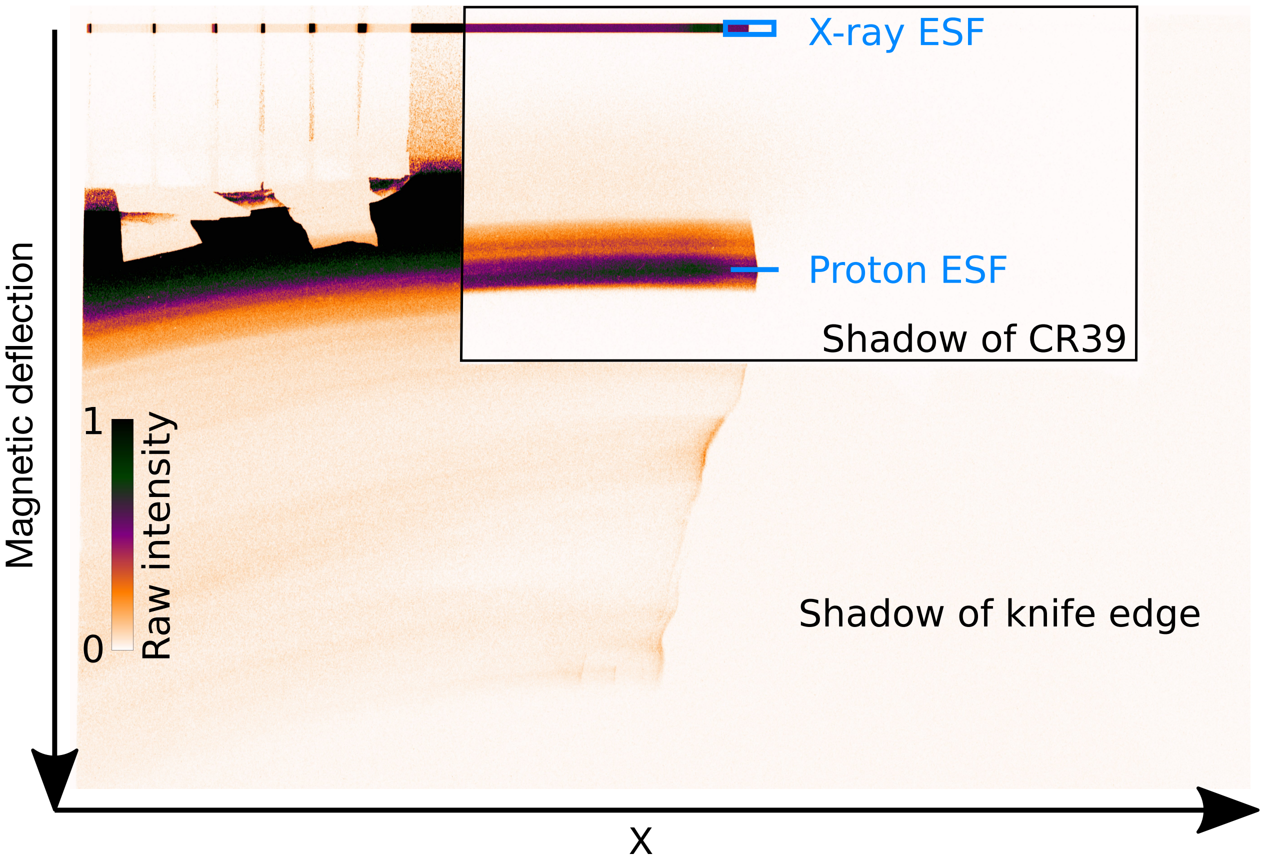

Supplementary Figure 4 | Measured detector raw data with sketch of measurements. *The edge spread function (ESF) for x-rays is measured in the direct projection of the slit, the ESF of protons is measured via a lineout close to the spectral peak. The CR39 ensures that neither neutral particles (in case of the x-rays), nor heavier ion beam contaminants (in case of the protons) perturb the results of spectral and ESF measurements. The sideward deflection of the protons likely stems from charge (variations) on the silicon edge. The source size for protons is therefore evaluated in a narrow energy range around the peak. Figure reproduced with permission from "Relativistically Intense Laser Microplasma Interactions" by Tobias Ostermayr, Springer Thesis, (2019).*

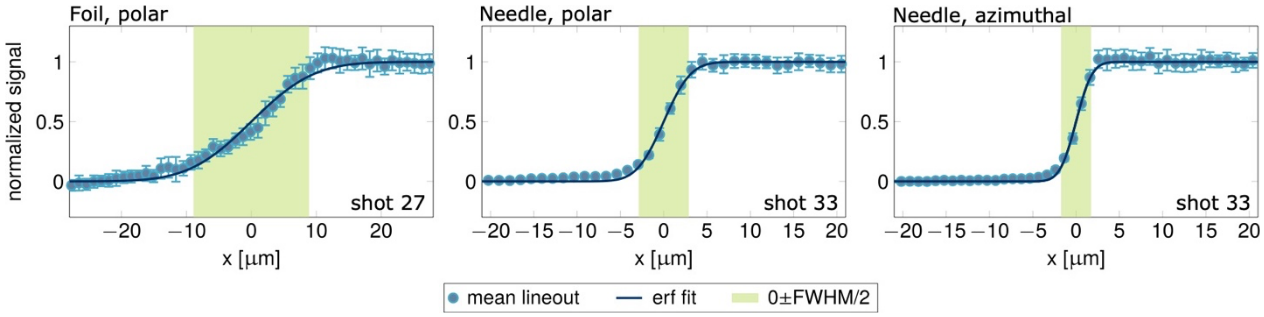

Supplementary Figure 5 | ESF measured for x-rays. *The fit to retrieve the source-size from the ESF neglects coherent effects and assumes an underlying Gaussian point spread function correspondingly assuming that an error function as a good fit. From this the FWHM is extracted and indicated by the green bands. Error bars show +/- SD of the measured signal.*

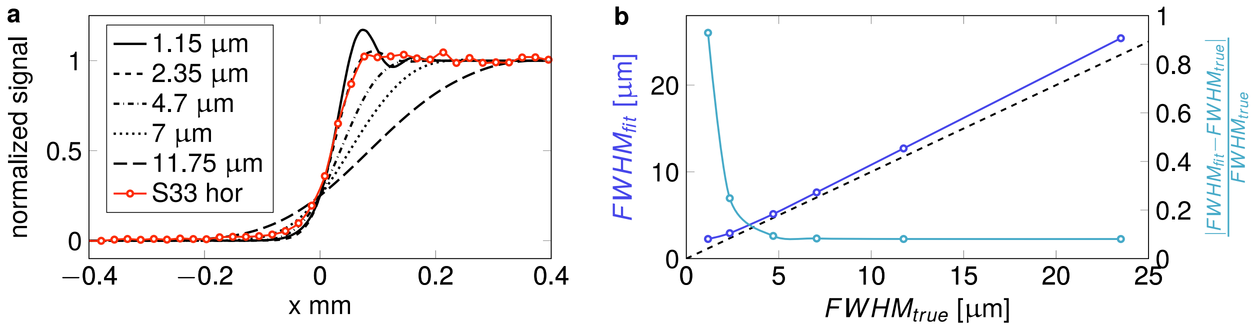

Supplementary Figure 6 | Considering diffraction in ESF measurements. *a: Calculation for x-rays considering diffraction (i.e. coherent effects) on the knife edge for various source size compared to our measurement (red). The calculation takes into account the spectrum from fig. 1b. This indicates the true effective source size around 2.35 µm. b: The relative error introduced by approximating the ESF with an error function (assuming a Gaussian source distribution and neglecting coherence) is small for source sizes larger than 2 µm.*

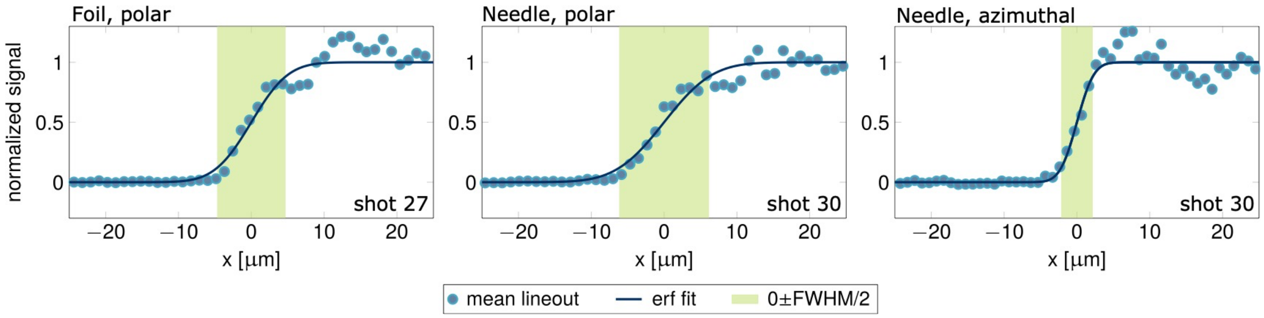

Supplementary Figure 7 | ESF measured for protons. *ESF measured for protons and evaluated using the same error function fit method.*

Supplementary Note 3: Transmitted laser

The transmitted laser pulse is recorded on a screen and indicates whether a substantial target interaction has been achieved. This confirms that non-depleted laser energy is mostly transmitted in the original laser propagation cone, even in successful shots with target interaction. In order not to destroy nearby imaging samples/objects with this transmitted laser pulse, the geometric separation of the laser pulse from the particle beams used for radiography is required.

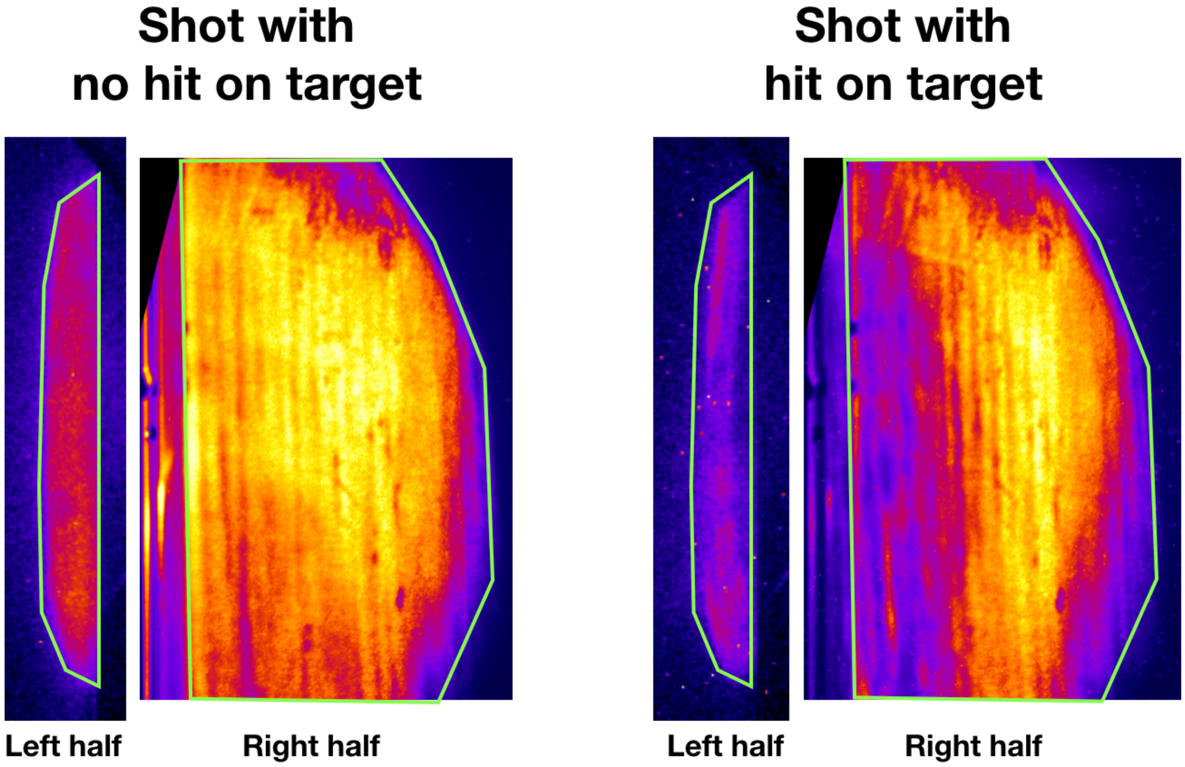

Supplementary Figure 8 | Transmitted laser beam profiles. *The transmission screen is split into two horizontal parts separated by 5 cm empty space in the center due to experimental constraints. Both are positioned downstream of the focus at a similar distance as the focusing element (which itself is upstream of the focus). Each screen (left half and right half) monitors half of the transmitted laser beam profile in the near field. Since each screen is recorded by an individual camera from an individual viewport, the geometric projections are different; to guide the eye, we marked the visible outline of the unperturbed laser-beam-halfs in light green.*

Supplementary Note 4: Additional Particle-in-Cell Simulation Plots

Additional simulations and data for Particle-in-Cell simulations are discussed in this supplementary note.

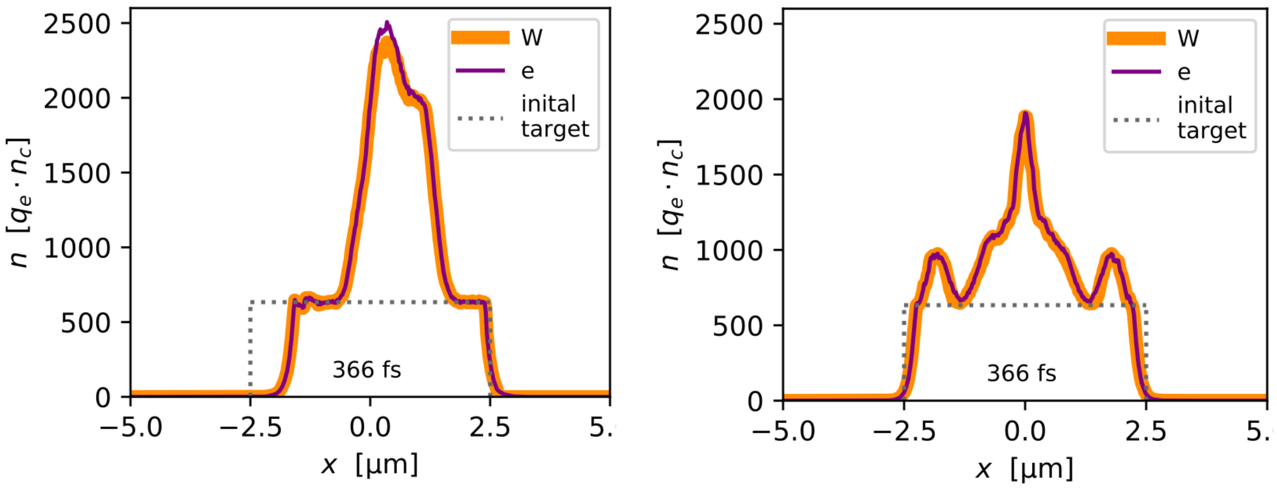

Supplementary Figure 9 | PIC simulation with s-polarization. *Central lineouts of the tungsten and electron density for a 2D3V PIC simulations with s-polarization. The left figure represents a slice along the laser axis and the right figure slices perpendicular through the target center. Data is presented two pulse lengths after peak intensity on target.*

2D3V modeling in s-polarization underestimates heating effects in the target, especially visible as the target rear is not expanding from its initial profile (contrary to Figure 1e in the main text). With significantly suppressed heating in this geometry, the density increase at the target front due to target hole boring and following compression of the target size results in an even smaller source size than the presented compression dynamics in p-polarization. The perpendicular lineout on the right shows little target radius reduction from the sides - due to the same missing heating effects - and a strong peak in the center from the longitudinal density compression.

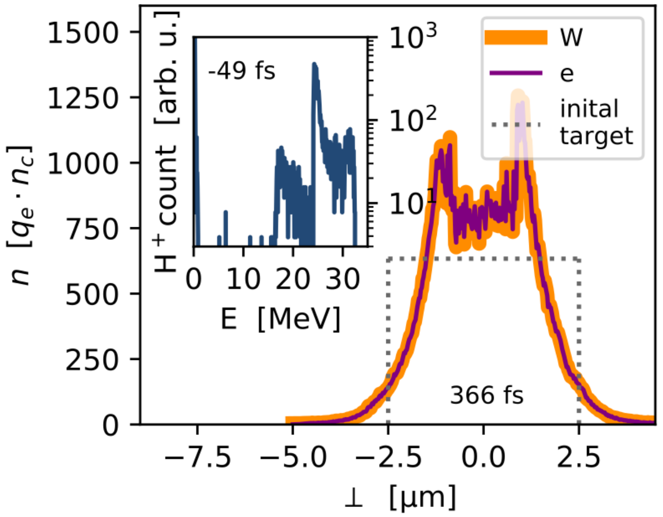

Supplementary Figure 10 | PIC simulation with p-polarization, perpendicular lineout. *This lineout complements figure 1e in the main text and shows overall target symmetric compression, leading to reduced effective source size.*

Supplementary figure 10 complements the longitudinal lineout in figure 1e of the main text with a perpendicular lineout through the target for the same point in time. In p-polarization, the target is also

compressed significantly from the sides to an overall symmetric compression to approximately 3 microns radius (from the original 5 micron needle radius).
